# Effect of Annealing Temperature on the Microstructure and Mechanical Properties of CoCrFeNiNb_0.2_Mo_0.2_ High Entropy Alloy

**DOI:** 10.3390/ma16113987

**Published:** 2023-05-26

**Authors:** Rui Fan, Sicong Zhao, Liping Wang, Lei Wang, Erjun Guo

**Affiliations:** 1 School of Material Science and Chemical Engineering, Harbin University of Science and Technology, Harbin 150000, China; 2 School of Mechanical and Electrical Engineering, Qiqihar University, Qiqihar 161000, China; 3 Key Laboratory of Advanced Manufacturing and Intelligent Technology (Ministry of Education), Harbin University of Science and Technology, Harbin 150000, China; 4 The Engineering Technology Research Center for Precision Manufacturing Equipment and Industrial Perception of Heilongjiang Province, Qiqihar 161000, China; 5 The Collaborative Innovation Center for Intelligent Manufacturing Equipment Industrialization, Qiqihar 161000, China

**Keywords:** high entropy alloy, annealing, nano-scale precipitate, mechanical properties

## Abstract

Strengthening the CoCrFeNi high entropy alloy with a face-center cubic structure has become a research prospect in the last decade. Alloying with double elements, Nb and Mo, is an effective method. In this paper, to further enhance the strength of the Nb and Mo contained high entropy alloy, CoCrFeNiNb_0.2_Mo_0.2_ was annealing treated at different temperatures for 24 h. As a result, a new kind of Cr_2_Nb type nano-scale precipitate with a hexagonal close-packed structure was formed, which is semi-coherent with the matrix. Moreover, by adjusting the annealing temperature, the precipitate was tailored with a considerable quantity and fine size. The best overall mechanical properties were achieved in the alloy annealed at 700 °C. The yield strength, ultimate tensile strength, and elongation are 727 MPa, 1.05 GPa, and 8.38%, respectively. The fracture mode of the annealed alloy is a mixture of cleavage and necking-featured ductile fracture. The approach employed in this study offers a theoretical foundation for enhancing the mechanical properties of face-centered cubic high entropy alloys via annealing treatment.

## 1. Introduction

Studies on high-entropy alloys (HEAs) have been prosperous for several decades since they were proposed in 2004 [1,2]. Among them, CoCrFeNi HEA with a face-centered cubic (FCC) structure is widely concerned due to its unique characteristic, such as thermal stability [3,4,5,6], corrosion-resistant [7,8,9], and high ductility [10,11]. However, the relatively low strength at room temperature limits their application prospects. To address this issue, a variety of strengthening mechanisms have been adopted, including grain refinement [12,13] and precipitation strengthening [14,15,16,17,18,19,20,21,22,23,24,25,26,27,28,29,30].

In particular, the precipitate strengthening HEAs have attracted great attention due to superior mechanical properties and interesting deformation behaviors. In order to enhance the strength of FCC HEAs, various efforts have been undertaken to introduce precipitate-forming elements. At present, most research is concerned with single-strengthening alloying element FCC HEAs. Alloying CoCrFeNi HEA with Al [14,15,16,17] can prompt the emergence of a body-centered cubic (BCC) phase within the pre-existing FCC matrix. The FCC crystal structure would undergo a transformation to BCC upon increasing the Al content. This transition could potentially enhance the strength of the HEA. However, it may also cause a decrease in ductility. When CoCrFeNi is alloyed with Ti [28,30], the resulting material undergoes a phase evolution. Specifically, as the molar content of Ti increases from 0 to 0.5, there is a transformation in the phase formation from FCC to FCC + σ + R and eventually to FCC + Laves + σ + R. The addition of Ti to FCC HEAs resulted in an increase in compressive strength from 871 MPa to 1502 MPa. However, this improvement came at the expense of a decrease in fracture strain from 75% to 20%. Moreover, it is observed that refractory alloying elements, such as Nb, Mo, V, Ta, and W, make HEAs more suitable for high-temperature applications but rob away their tensile strength while increasing compressive strength [31]. This may be due to the precipitates in these HEAs tends to be relatively bulky, which would limit the precipitate strengthening effect. Thus, for a wider range of applications, achieving a balance of properties between compressive and tensile strength is one of the challenges for researchers. In recent studies conducted by He [29,32], Xu [33], and Fu [34] et al., the strength of FCC HEAs has been significantly enhanced through a novel approach of combined alloying with Al and Ti. This method promotes the formation of L1_2_, hierarchical intragranular γ*, and γ′ phases within the matrix, similar to the strengthening mechanisms employed in superalloys. These results demonstrate a remarkable balance between strength and plasticity, indicating their potential for use in various high-performance applications. The incorporation of double elements has proven to be a promising strategy for improving the mechanical properties of FCC HEAs.

Following this line, we introduced Nb and Mo as alloying elements to CoCrFeNi, resulting in high-performance HEAs. The addition of Mo and Nb led to the formation of a Fe2Nb-type Laves phase rich in these elements, which precipitated in the FCC matrix. This microstructure resulted in exceptional strength and plasticity properties for the alloy [35]. However, the size of precipitates in the as-cast alloy is still relatively large. The introduction of fine precipitates into the alloy has the capability to further increase the strength of the HEAs. In addition to reasonable composition design, heat treatment is one of the effective methods to induce fine precipitates. For example, Shun et al. [36] enhanced the CoCrFeNiMo_0.85_ alloy through heat treatment at different temperatures. After annealing, a minor (Mo, Cr)-rich μ phase was induced into the alloy. Feng et al. [24] strengthened the CoCrFeNiNb_0.25_ using the annealing treatment. After annealing, an Nb-rich lath-shaped phase with FCC structure was formed in the matrix. In our investigation of Nb and Mo alloyed HEAs, during synthesis, the matrix forms a supersaturated solid solution due to rapid cooling. Therefore, we are curious to see if annealing can lead to the formation of fine precipitates and ultimately enhance the strength of the HEAs.

Inspired by this idea, our previously proposed CoCrFeNiNb_0.2_Mo_0.2_ HEA [35] was annealed at different temperatures to induce fine precipitate. The microstructure of as-annealed HEAs was characterized in detail, the relevance between microstructure and mechanical properties was discussed, and the fracture mechanism was analyzed.

## 2. Materials and Methods

The ingots of CoCrFeNiNb_0.2_Mo_0.2_ HEA were synthesized in a WK-II type non-consumable vacuum arc-melting furnace by the mixture of pure elements, which purity is larger than 99.9 wt%. The smelting current is 500A. The alloy was melted by arc in an atmosphere of high-purity argon, which had been treated with titanium to remove impurities. The melting process was conducted in a copper crucible with water cooling. Each ingot was remelted five times to ensure homogeneity. The quantity of each ingot is ~100 g. The samples were cut from the ingots by wire cutting parallel to the horizontal plane and annealed at temperatures ranging from 600 to 800 °C (denoted AN600, AN700, and AN800) for 24 h. The crystal structure was identified using X-ray diffraction (XRD) (X’Pert PRO, PANalytical B.V., Almelo, The Netherlands) equipped with Cu Kα at 40 kV and 40 mA. The X-ray diffractometer collects data at a constant interval of 5° of 2θ while scanning a range from 20° to 100°, during which the intensity of X-ray diffraction peaks is recorded. The samples for XRD were first ground to smooth with silicon carbide papers, then polished with diamond polishing paste to get a mirror-like surface. The microstructure and chemical compositions were characterized by a scanning electron microscope (SEM) (Apreo C, Thermo Fisher Scientific Inc., St. Bend, OR, USA) equipped with an energy-dispersive X-ray spectrometer (EDS). To prepare the samples for SEM characterization, a multi-step process was followed. First, the samples were ground with silicon carbide papers to remove any roughness. Then, the diamond polishing paste was used to polish the samples. Finally, the polished surface was etched using an aqua-regia solution for 10 s. The formation of phases and interface between the matrix and precipitate were examined using a transmission electron microscope (TEM) (FEI Tecnai G2 F30, FEI Company, Hillsboro, OR, USA) operating at 300 kV. The TEM samples were prepared by grinding to a thickness of less than 50 μm, then ion-milling at a 5 kV ion gun energy and 4° milling angle. Additionally, tensile tests at room temperature were performed using a universal tensile machine (MTS E44.304, MTS Systems Co., Eden Prairie, MN, USA). The tensile rate was 1 mm/min. The gauge section of dog-bone-shaped specimens used for the tensile test was 6 mm in length, 2 mm in width, and 1 mm in thickness. The number of samples for the tensile test was three per annealing temperature. The dimension and volume fraction of each phase were measured and calculated by Photoshop 2018 and Image-Pro Plus 6.0 software.

## 3. Results and Analysis

### 3.1. Crystal Structure

**Figure 1:**
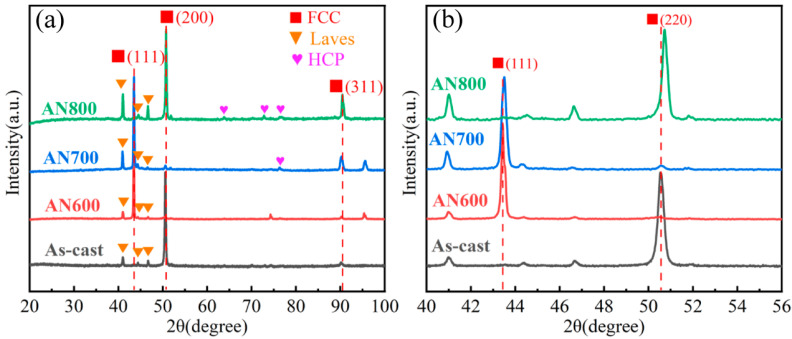
(**a**) XRD patterns of the as-cast HEA, AN600, AN700, and AN800, (**b**) Magnification for the (111)_FCC_ and (200)_FCC_ peaks.

The XRD patterns of the researched HEAs are presented in Figure 1a. In AN600, only peaks of Laves phase and FCC matrix can be detected, which is the same with the as-cast alloy. In AN700, an extra peak was detected, while more extra peaks were detected in AN800. Through comparison, these extra peaks may correspond to the hexagonal close-packed (HCP) structured Cr_2_Nb phase. The lattice parameters of the HCP phase obtain from XRD are a = 4.97 Å and c = 8.05 Å. Furthermore, in comparison to the as-cast HEA, the peaks associated with the FCC matrix display a noticeable shift towards higher 2θ angles upon annealing, signifying a reduction in the lattice parameters of the matrix. This phenomenon is particularly evident for the (111) and (200) peaks, as illustrated in Figure 1b. After annealing, the lattice constant of the matrix would decrease due to the precipitation of Nb and Mo with a large atomic radius, which may be the reason for the peak shift.

### 3.2. Microstructures

**Figure 2:**
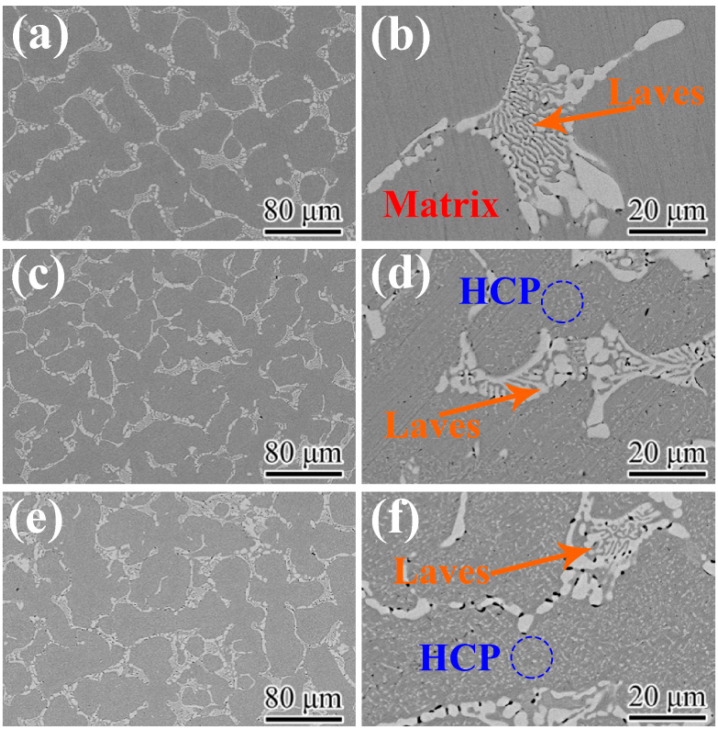
BSE-SEM images of the as-annealed HEAs: (**a**,**b**) AN600; (**c**,**d**) AN700; (**e**,**f**) AN800.

**Figure 3:**
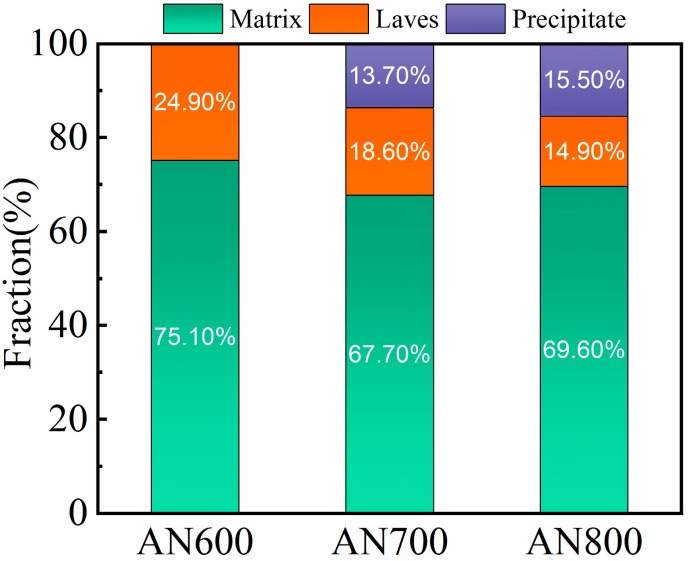
Volume fractions of the matrix, Laves phase, and precipitate in as-annealed HEAs.

In order to further identify the newly precipitated phase corresponding to the extra peaks of XRD patterns, the backscatter SEM (BSE-SEM) images of the microstructures of the as-annealed HEAs are displayed in Figure 2. The low magnification morphologies are shown in Figure 2a,c,e. The detailed high magnifications are shown in Figure 2b,d,f. The low magnification images reveal that the microstructures of the HEAs are primarily comprised of two phases, FCC matrix and Laves phase. As shown in Figure 2b, except for FCC matrix and Laves phase, no other new phase can be observed in AN600. A large amount of lamellar structures of the Laves phase were preserved, indicating that the Laves phase has good thermal stability at 600 °C. In AN700, a considerable amount of fine precipitates can be observed besides FCC matrix and Laves phase, as shown in Figure 2d (marked by a blue circle). The precipitate corresponds to the HCP phase detected by XRD. Moreover, for the Laves phase, the lamellar structure became coarser and less numerous. As shown in Figure 2f, more HCP phase was precipitated, and the amount of the lamellar structure was further reduced. The statistics of volume fractions of each phase formed in the matrix are shown in Figure 3. The as-annealed HEAs have varying volume fractions of Laves phase, with values of 24.9%, 18.6%, and 14.9%, respectively. In addition, the volume fractions of the HCP phase also differ among the HEAs, with percentages of 0%, 10.7%, and 15.5%, respectively.

**Figure 4:**
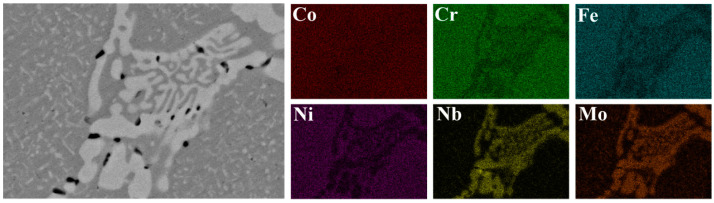
EDS mapping of Laves phase in AN800.

**Figure 5:**
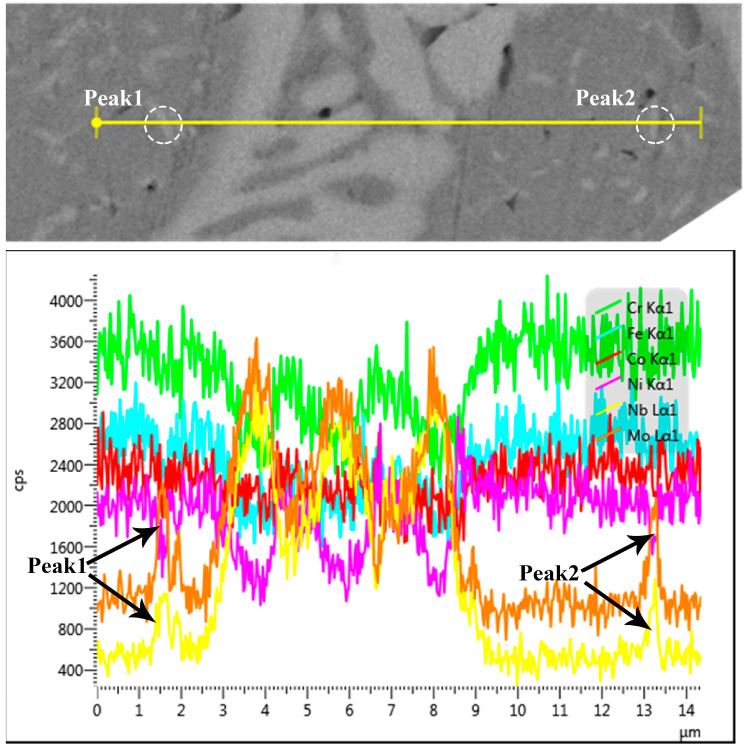
EDS line scanning of HCP precipitate.

The EDS mapping of Laves phase in AN800 are shown in Figure 4. The results reveal a homogeneous distribution of Co, Cr, Fe, and Ni within the matrix. Moreover, the Laves phase displays a noticeable depletion in Cr, Fe, and Ni while exhibiting an enrichment in Nb and Mo. In order to identify the chemical compositions of the HCP precipitate, EDS line scanning of AN700 was conducted, as shown in Figure 5. The results reveal that the HCP precipitate is enriched in Nb and Mo as shown by the peaks marked by arrows. Table 1 presents the results of the EDS point analysis conducted on AN700 in order to characterize the chemical constituents of the various phases present in the investigated HEAs. The analysis reveals that the recently formed fine precipitate exhibits a significant enrichment in Nb and Mo. The ratio of Co, Cr, Fe, and Ni elements to Nb and Mo elements is close to 2:1. Combined with the XRD results, the fine precipitate is a Cr2Nb-type HCP phase. Furthermore, when the annealing temperature increased, the quantity of the precipitate increased. The precipitation of solute elements, Nb and Mo, will result in the decrease in lattice parameters of the FCC matrix, which explains why, in XRD patterns, the peaks corresponding to the FCC matrix phase shift to a higher 2θ when the annealing temperature increased. Additionally, Ostwald ripening is the underlying mechanism driving the coarsening of the lamellar-structured Laves phase [37]. That is, smaller precipitates make the system have higher interfacial energy. In order to reduce the total interfacial energy, the fine precipitates with high density tend to be coarsened into large ones with a smaller total interface and low-density distribution.

**Figure 6:**
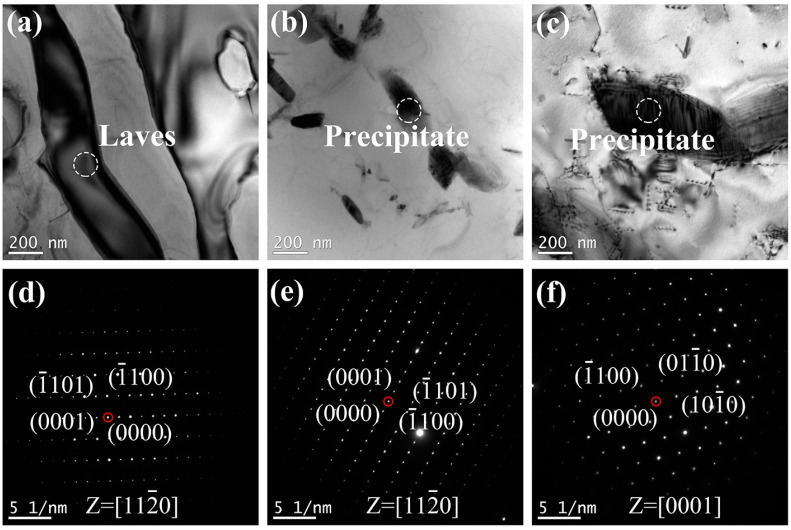
The bright field TEM images of (**a**) AN600, (**b**)AN700, (**c**)AN800; SAED patterns corresponding to (**d**) Laves phase in AN600, (**e**) precipitate in AN700, and (**f**) precipitate in AN800.

In order to further investigate the microstructure of the HCP precipitate, the researched HEAs were observed by TEM. The bright field TEM images of AN600, AN700, and AN800 are shown in Figure 6a–c. The corresponding selected area electron diffraction (SAED) patterns are shown in Figure 6d–f. In AN600, as shown in Figure 6a, except for a micron-scale phase, there was no other second phase precipitated. The micron-scale phase is identified to be Laves phase from the SAED pattern (Figure 6d) in AN600 taken along the $[11\overset{-}{2}0]$ zone axis, which is consistent with the results of XRD and SEM. As shown in Figure 6b, a nano-scale precipitate was formed in the matrix, and the average dimension of the precipitate is 95 nm. The nano-scale precipitate is indexed as a Cr_2_Nb type phase with an HCP structure from the SAED pattern obtained along $[11\overset{-}{2}0]$ zone axis, as shown in Figure 6e. The lattice parameters of the new HCP phase are a = 0.4610 nm and c = 0.7497 nm. As shown in Figure 6c, the size of the precipitate increased. The average dimension of the precipitate is 310 nm. The corresponding SAED pattern (Figure 6f) taken along [0001] reveals that the precipitate is also the Cr_2_Nb type phase. As the annealing temperature increases, both the size and the volume fraction increase, which is because that higher temperature would provide sufficient impetus for precipitation.

**Figure 7:**
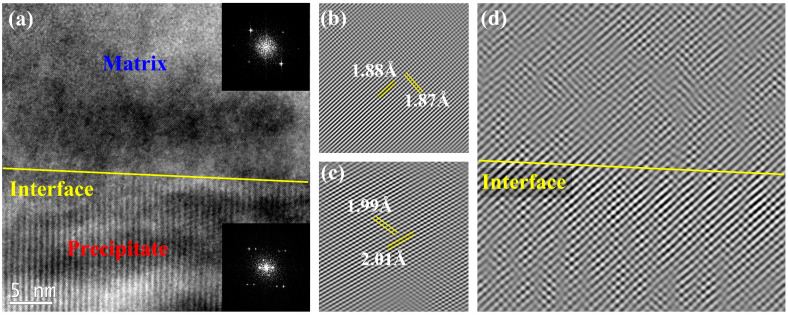
(**a**) The HRTEM image of the interface between FCC matrix and HCP precipitate, (**b**,**c**) the IFFT image of FFT inset in (**a**) corresponding to FCC matrix and HCP precipitate, respectively, (**d**) the enlarged interface between the matrix and precipitate.

As shown in Figure 7a, the interface between the HCP precipitate and matrix is presented in the high-resolution transmission electron microscope (HRTEM) image. Figure 7b,c show the inverse fast Fourier transform (IFFT) images of the FFT inset in Figure 7a corresponding to the HCP precipitate and FCC matrix. These images correspond to the HCP precipitate and matrix, respectively. The distances between crystal planes $(\overset{-}{2}\overset{-}{1}1)$ and $(1\overset{-}{1}1)$ of the FCC matrix are 1.88 Å and 1.87 Å, respectively, while those for $(0\overset{-}{1}11)$ and $(1\overset{-}{1}01)$ planes of HCP precipitate are 2.01 Å and 1.99 Å. As presented in Figure 7d, the interface between the matrix and HCP precipitate is semi-coherent. Combined with its fine size and considerable volume fraction, the precipitate has an excellent strengthening effect.

### 3.3. Mechanical Properties and Relevance to Microstructure

**Figure 8:**
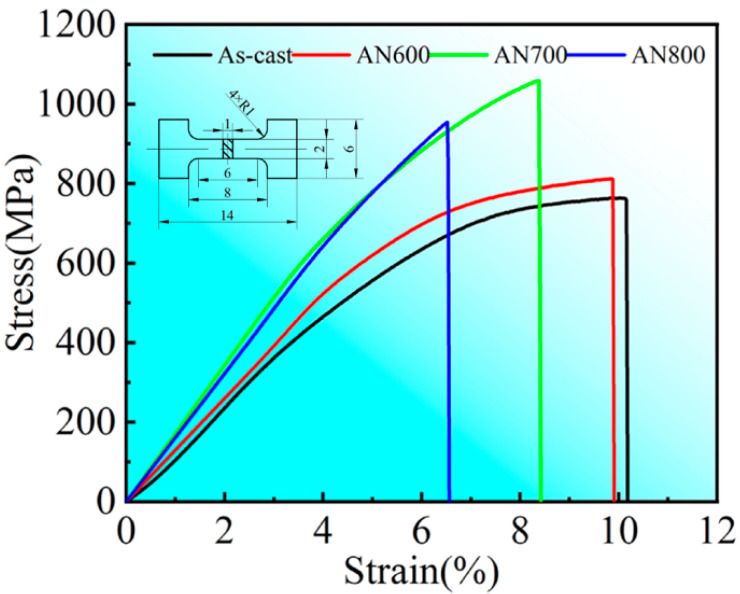
Stress–strain curves of the as-cast CoCrFeNiNb_0.2_Mo_0.2_, AN600, AN700, and AN800 HEAs.

**Figure 9:**
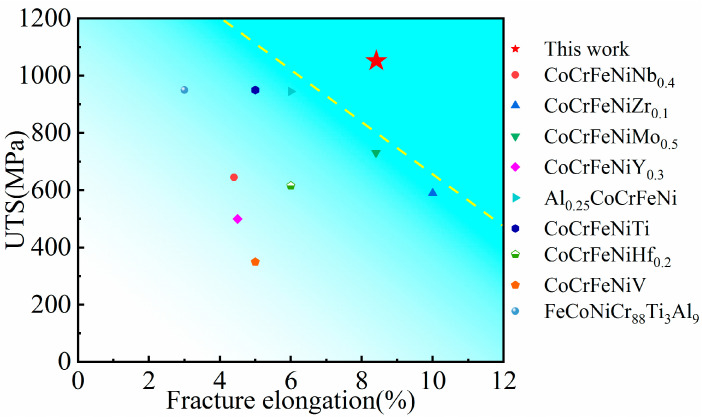
Comparison of the UTS and fracture elongation of the current HEA with those of other CoCrFeNi-based HEAs.

Tensile tests at room temperature were conducted to investigate the strengthening effect of the precipitate formed after annealing. The engineering stress–strain tensile curves for the as-cast and annealed HEAs are shown in Figure 8, and the results are listed in Table 2. The results show that the strength of the researched HEA could be enhanced by annealing treatment due to the precipitation of a considerable amount of nano-scale HCP phase, which is semi-coherent with the matrix. The best overall tensile properties were achieved in AN700, with a YS of 727 MPa, UTS of 1.05 GPa, and elongation of 8.38%. A comparison of the UTS and elongation of the current HEA with those of other CoCrFeNi-based HEAs [25,26,27,28,29,38,39,40] is presented in Figure 9. The AN700 HEA in this work is located at the upper-right above the conventional HEAs, indicating that its tensile properties outperform most current FCC-based HEAs. Compared to the as-cast HEA, the UTS of AN700 increased by nearly 40%, while the elongation decreased by only less than 2%. The excellent strengthening effect is due to the dispersion of the nano-scale HCP precipitate, which is semi-coherent with the matrix. The tensile properties of AN600 changed little because the annealing temperature is not high enough to provide sufficient impetus for precipitation leading to the microstructure varying little. In AN800, the volume fraction of the precipitate is the largest. However, the size of the precipitate increased a lot, and the lamellar structure of Laves phase became coarser due to the Ostwald ripening mechanism, which would lead to the reduction in the plasticity. As a result, the tensile specimen fractured just past the yield point due to the alloy embrittlement.

### 3.4. Fracture Morphologies

**Figure 10:**
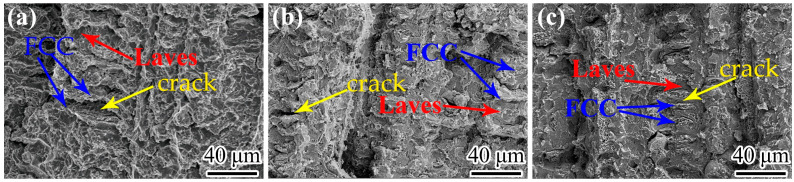
The fracture surface morphologies of (**a**) AN600, (**b**) AN700, and (**c**) AN800 HEAs.

To gain a deeper understanding of the fracture mechanism and the interplay between microstructure and mechanical properties, SEM was utilized to examine the fracture morphologies of the HEAs, as illustrated in Figure 10. The fracture behavior of the AN600, AN700, and AN800 HEAs exhibited two distinct modes for the Laves phase and FCC matrix phase, as illustrated in Figure 10a–c. The Laves phase exhibited a cleavage fracture mode, which is a brittle fracture mechanism characterized by the formation of a flat and smooth surface. In contrast, the FCC matrix phase demonstrated ductile fracture morphology, characterized by the occurrence of necking and the formation of sharp fracture lines, without the formation of characteristic dimples typically observed in ductile materials. The phase boundaries between the FCC and Laves phases were found to be the site of crack initiation, where stress concentration led to crack nucleation. Subsequently, the crack propagated into the Laves phase. Additionally, compared to the as-cast HEA [35], the annealed HEA has a stronger matrix due to the precipitation of a fine second phase. However, this strengthening comes at the cost of slightly decreased plasticity. Fracture morphology analysis reveals that the area corresponding to the FCC matrix is flatter in the annealed HEAs.

## 4. Conclusions

In this investigation, we tailored the microstructure of CoCrFeNiNb_0.2_Mo_0.2_ by adjusting the annealing temperature to further enhance the strength of the HEA. The following conclusions can be achieved from experimental results:

(1) After annealing treatment at an appropriate temperature, the phase formation of the researched HEA transformed from Laves + FCC to Laves + HCP + FCC. The dimension and the volume fraction of Laves phase and the newly precipitated HCP phase would be various when the annealing temperature changed. Both the Laves phase and HCP precipitate would be coarser as the temperature increase.
(2) A new kind of Cr_2_Nb type HCP precipitate was formed after annealing, which is semi-coherent with the matrix and has an excellent strengthening effect on the current HEA system.
(3) The microstructure of HEA was tailored by adjusting the annealing temperature. A considerable amount of nano-scale precipitate was formed, leading to an enhancement of the mechanical properties. The best overall tensile properties were achieved in AN700. The YS, UTS, and elongation are 727 MPa, 1.05 GPa, and 8.38%, respectively.
(4) After annealing, the materials AN600, AN700, and AN800 exhibit fracture patterns that comprise both cleavage and ductile fractures in their respective phases of Laves and FCC. The ductile fracture displays a necking feature rather than the presence of typical dimples. Cracks are observed to initiate at the boundary between the two phases due to localized stress concentration and subsequently propagate within Laves phase. The presence of nano-scale HCP phase precipitation results in a slight reduction in matrix plasticity and a flatter fracture area in the FCC matrix.

## Figures and Tables

**Table 1:** EDS results (at.%) of AN700 HEA.

Phase	Co	Cr	Fe	Ni	Nb	Mo
Nominal	22.73	22.73	22.73	22.73	4.54	4.54
Matrix	22.72	24.73	23.71	24.96	1.48	2.38
Laves	22.07	18.80	16.78	13.44	17.33	11.57
HCP	17.94	20.62	16.26	8.23	6.27	30.68

**Table 2:** Tensile properties of as-cast and as-annealed HEAs.

Alloys	YS(MPa)	UTS(MPa)	Elongation (%)
As-cast	510 ± 5	763 ± 7	10.0 ± 0.31
AN600	572 ± 4	814 ± 7	9.91 ± 0.27
AN700	727 ± 6	1050 ± 10	8.38 ± 0.20
AN800	756 ± 10	954 ± 10	6.56 ± 0.17

## Data Availability

The data presented in this study are available on request from the corresponding author.
